# Evaluating the Psychological Impacts Related to COVID-19 of Vietnamese People Under the First Nationwide Partial Lockdown in Vietnam

**DOI:** 10.3389/fpsyt.2020.00824

**Published:** 2020-09-02

**Authors:** Xuan Thi Thanh Le, Anh Kim Dang, Jayson Toweh, Quang Nhat Nguyen, Huong Thi Le, Toan Thi Thanh Do, Hanh Bich Thi Phan, Thao Thanh Nguyen, Quan Thi Pham, Nhung Kim Thi Ta, Quynh Thi Nguyen, Anh Ngoc Nguyen, Quan Van Duong, Men Thi Hoang, Hai Quang Pham, Linh Gia Vu, Bach Xuan Tran, Carl A. Latkin, Cyrus S. H. Ho, Roger C. M. Ho

**Affiliations:** ^1^Institute for Preventive Medicine and Public Health, Hanoi Medical University, Hanoi, Vietnam; ^2^Office of Inspector General Department, U.S. Environmental Protection Agency, Atlanta, GA, United States; ^3^Institute for Global Health Innovations, Duy Tan University, Da Nang, Vietnam; ^4^UFR Biosciences Department, Université Claude Bernard Lyon 1, Villeurbanne, France; ^5^Faculty of Medicine, Duy Tan University, Da Nang, Vietnam; ^6^Center of Excellence in Evidence-based Medicine, Nguyen Tat Thanh University, Ho Chi Minh City, Vietnam; ^7^Bloomberg School of Public Health, John Hopkins University, Baltimore, MD, United States; ^8^Department of Psychological Medicine, National University Hospital, Singapore, Singapore; ^9^Department of Psychological Medicine, Yong Loo Lin School of Medicine, National University of Singapore, Singapore, Singapore; ^10^Institute for Health Innovation and Technology (iHealthtech), National University of Singapore, Singapore, Singapore

**Keywords:** national partial lockdown, social isolation, psychological impacts, COVID-19, Vietnam

## Abstract

This is the first time in Vietnam that people have undergone “social distancing” to minimize the spreading of infectious disease, COVID-19. These deliberate preemptive strategies may have profound impacts on the mental health of the population. Therefore, this study aimed to identify the psychological impacts of COVID-19 on Vietnamese people and associated factors. We conducted a cross-sectional study during a one-week social distancing and isolation from April 7 to 14, 2020, in Vietnam. A snowball sampling technique was carried out to recruit participants. Impact of Event Scale-Revised (IES-R) was utilized to assess the psychological impacts of the COVID-19. Of all participants, 233 (16.4%) reported low level of PTSS; 76 (5.3%) rated as moderate, and 77 (5.4%) reported extreme psychological conditions. Being female, above 44 years old, or having a higher number of children in the family were positively associated with a higher level of psychological distress. Being self-employed/unemployed/retired was associated with a higher score of intrusion and hyperarousal subscale. Individuals who have a history of touching objects with the possibility of spreading coronavirus (utensils) were related to a higher level of avoidance. There were relatively high rates of participants suffering from PTSS during the first national lockdown related to COVID-19. Comprehensive strategies for the screen of psychological problems and to support high-risk groups are critical, especially females, middle-aged adults and the elderly, affected laborers, and health care professionals.

## Introduction

The 2019 coronavirus disease (COVID-19) pandemic is a global health threat. The number of cases continues to escalate exponentially beyond China, spreading to over 210 countries and territories (1). Clusters of unknown pneumonia cases were first detected in late December 2019, with epidemiological exposure linked to a seafood market in Wuhan, China (2). This pandemic has sown the seeds of an unprecedented global looming crisis, especially in developing countries, where health systems are fragile (3) and lack the capacity to impose restrictions or offer social assistance net for the unemployed (4).

Because of COVID-19, Vietnam, a developing country, was on higher alert due to its land border with China and overseas travel between two countries related to business and tourism. The government of Vietnam imposed “social distancing and social isolation” at the beginning of April 2020 to mitigate the spread of COVID-19, with prompt contact tracing and quarantine (5). Social distancing, or “physical distancing,” means that one person keeps a safe space (about 2 arms’ length) from other people who are not from their household in both indoor and outdoor spaces. For the first time in Vietnam, people have undergone “social distancing” to minimize infectious disease transmission. Although, these deliberate preemptive strategies bring positive effects on slowing down of positive tests, abiding by social distancing may ramp up profound impacts on the mental health of the population (6). At the individual level, mass home-confinement directives (quarantine or self-isolation) are associated with numerous adverse emotional outcomes such as depression, boredom, irritability, and stigma related to quarantine (7). Emotional distress stressors include infringement of personal freedoms due to unfamiliar public health measures, unemployment resulting in financial losses, the fear of COVID-19 infection, and severe shortages of personal protective equipment (7–9). As the government places stringent social restrictions, the people also have to confront a pervasive impairment of society. The loss of meaningful connections, lack of education due to school closures among children, and difficulty in ensuring safe medical care of the elderly can increase the risk of psychiatric illness attributed to COVID-19 (10–12).

The COVID-19 pandemic has triggered direct and indirect psychological effects on people over the world. A previous study revealed that the imposition of lockdown in China put more than 50 million people under quarantine to prevent the infection, leading to a “desperate plea” for support (13). More than half of the participants rated the pandemic’s psychological impact as moderate or severe, and about one-third of them reported moderate to severe anxiety symptoms (13). Research studies from Iran and Japan also highlighted the seriousness of the COVID-19, misinformation, social isolation resulting in mental health problems, and a high level of panic behavior, such as stockpiling of resources in the population (14, 15). The severe acute respiratory syndrome (SARS) epidemic in 2003 was positively related to a high level of anxiety among recovered patients and the risk of suffering from post-traumatic stress disorder among those who survived life-threatening condition (16, 17). In the H1N1 influenza outbreak, the general public also revealed fears about the probability of contracting the virus (11). Lessons learned from previous epidemics show that assessment and interventions play a critical role in mitigating the psychological issues (18).

In countries with a large number of COVID-19 cases and deaths, many people are aware that lockdown is essential to reduce the risk of COVID-19 infection and its consequences. In Vietnam, however, the number of cases has only reached 271 [20]. There have been 252,950 deaths globally, though Vietnam has zero deaths, which may cause a false sense of safety among the country’s citizens (19). As a result, restrictive measures to achieve public health goals could trigger adverse reactions due to differences in acceptability, especially those who perceived lack of threat (20). According to social distancing and isolation, all citizens were recommended to stay at home. They could only go out for essential services, such as food, medicine, emergencies, working at establishments that cannot work from home, and other emergency cases. The citizens were required to keep an interpersonal distance (2 m) and limit public gatherings to no more than two people. In addition, the epidemic is also occurring against the backdrop of growing mental health issues in particularly vulnerable groups in Vietnam (21). Previous studies suggested that people with different social-economic backgrounds and history/risk of exposure to the COVID-19 experienced a different severity level of mental health problems (13, 22, 23). Therefore, this study aimed to identify the psychological impacts of COVID-19 on Vietnamese people under the first nationwide lockdown. The results may help us develop strategies to mitigate psychological effects and enhance the ability to respond to future epidemics and pandemics.

## Materials and Methods

### Study Setting and Participants

#### Study Design

We conducted a cross-sectional study during a one-week social distancing and isolation from April 7 to 14, 2020, in Vietnam.

#### Research Population and Inclusion Criteria

Participants of this study were the Vietnamese people and chosen based on the following eligibility criteria:

- Living in Vietnam since the first COVID-19 case in Vietnam was detected at the end of January 2020
- Agreeing to participate in the study by approving the online informed consent
- Can access the online questionnaire on the web-based platform
- Not experiencing an acute medical condition/physical impairments or be emergency

### Sample Size and Sampling Method

A snowball sampling technique was carried out to recruit participants. Snowball sampling refers to a chain-referral sampling and a nonprobability sampling technique, which involved participants offering referrals to recruit other subjects required for the study (24). In the beginning, a primary data source will be selected and then nominate other potential data sources that can take part in the survey. This technique is extensively utilized when the study population is unknown, and it is tough to select participants meeting the eligibility criteria (24). Thus, in our study, snowball sampling was the most appropriate technique to recruit participants as we were not able to retrieve official lists of members’ demographic information. Previous studies also used snowball sampling as a useful tool to assess the mental health problems of the different populations during the COVID-19 pandemic (25, 26).

The primary groups in this study were students, lecturers, and staff from Hanoi Medical University. The link of the survey was sent to participants of core groups *via* social networks and emails. They accessed the link *via* their laptops/tablets/smartphones and sent the research invitation to different groups by social networks and emails after finishing the survey. We encouraged participants to roll out the study to as many as possible. The study participants consisted of different socio-economic characteristics of gender, age, educational level, and occupation, including health care workers, professional educators, white-collar workers, and students from 63 provinces and cities of Vietnam. There were 1,423 participants involved in the research.

### Measure and Instruments

At the beginning of the survey, the introduction of research and informed consent were presented. After agreeing to involve in the study, participants answered a range of questions, including:

#### Socio-Economic Characteristics

The socio-economic characteristics included gender, age, educational level, marital status, occupation, religion, living region, number of children in the family.

#### History of Exposure to COVID-19 Infection

Participants self-reported their risk of exposure to COVID-19, which consisted of having COVID-19 confirmation testing or being isolated within 14 days, and the history of exposure to COVID-19-infected people.

#### Psychological Impacts

We utilized an easily administered self-report questionnaire, Impact of Event Scale-Revised (IES-R), to assess the psychological impacts of the COVID-19. In this study, this tool evaluated post-traumatic stress symptoms (PTSS) or acute stress and its severity level as immediate responses of participants within a short period of time after exposure to a specific traumatic event, not PTSD diagnostic (27). The questionnaire covered 22 questions and divided into three main post-traumatic symptoms (intrusion, avoidance, and hyperarousal). Each question was rated from 0 (Not at all) to 4 (Extremely). The score of Intrusion subscale ranged from 0 to 32 (eight items: 1, 2, 3, 6, 9, 14, 16, 20), Avoidance subscale ranged from 0 to 32 (eight items: 5, 7, 8, 11, 12, 13, 17, 22) and Hyperarousal subscale ranged from 0 to 24 (six items: 4, 10, 15, 18, 19, 21). The outcome of each subscale was analyzed as continuous measures. The total score was calculated as continuous measure which ranged from 0 to 88; higher scores indicated more PTSS. IES-R was also classified as normal (0–23), low level of PTSS (PTSD Clinical concern; 24–32), moderate level (a probable diagnosis of PTSD; 33–36), and extreme level (>37). The IES-R has been validated elsewhere in the Vietnam veterans with Cronbach’s alpha = 0.96, which assesses the reliability/internal consistency (28).

### Data Analysis

We used STATA 15.0 (StataCorp LP, College Station, TX) to analyze the data. Descriptive statistics presented included frequencies, percentages, means, and standard deviations.

We applied multivariable regression models to identify factors associated with the psychological impacts of participants during COVID-19. The outcomes of regression models were the total score of IES-R and three subscales (Intrusion, Avoidance, and Hyperarousal), which were measured as continuous variables. The independent variables included in the regression models were socio-economic characteristics and history of exposure to COVID-19 infection. Details of independent variables were shown in **Table 1**.

**Table 1:** Details of independent variables of regression models.

Variables	Types of variable	Compared group
Age	Ordinal	Under 25
Gender	Categorical	Male
Educational level	Ordinal	High school and below
Marital status	Categorical	Single
Occupation	Categorical	Health workers
Religion	Categorical	No
Living region	Categorical	Northern
Number of children in the family	Continuous	
Having COVID-19 confirmation testing	Binary	No
Being isolated within 14 days due to COVID-19	Binary	No
History of exposure to COVID-19-infected people	Categorical	No

To determine the reduced regression models, we used stepwise forward selection strategies. At first, we put all variables into the models. This technique alternated between forward and backward, which brought in and removed variables meeting the criteria for entry or removal until a stable set of variables was obtained. The p-value for the log-likelihood ratio test of the stepwise method was 0.2. A p-value of less than 0.05 was considered as statistically significant.

### Ethical Consideration

The Review Committee of the Institute for Preventive Medicine and Public Health, Hanoi Medical University, approved the study. Participants voluntarily took part in the survey, and the anonymities were secured. Participants could refuse or withdraw from the study at any time.

## Results

**Table 2** presents the socio-economic characteristics of the participants. Approximately two-thirds of the participants were female (n = 945 (66.4%)). The majority of the sample lived in the Northern region of Vietnam ((n = 1108 (77.9%)). More than half of participants reported undergraduate educational level (n = 801 (56.3%)), and 63.4% (n = 902) currently lived with their spouse/partner. Health care workers or professional educators made up 38% (n = 541) of participants. The mean age was 35.0 years old (SD = 11.0).

**Table 2:** Socio-economics characteristics of participants.

Characteristics	n	%
**Gender**		
Male	475	33.4
Female	945	66.4
Others	3	0.2
**Region**		
Northern	1,108	77.9
Central	165	11.6
Southern	139	9.8
Foreign	11	0.8
**Age group (years)**		
Under 25	337	23.7
25–34	380	26.7
35–44	378	26.6
Above 44	326	22.9
**Religion**		
Yes	244	17.2
No	1,179	82.9
**Marital status**		
Single	482	33.9
Living with a spouse/partner	902	63.4
Others	39	2.7
**Education level**		
High school and below	291	20.5
Undergraduate	801	56.3
Postgraduate	331	23.3
**Occupation**		
Health workers	256	18.0
Professional educators	285	20.0
White-collar workers	318	22.4
Students	302	21.2
Others	262	18.4
**Occupation status**		
Functionary	543	38.2
Unlimited term full-time contract	279	19.6
Limited-term full-time contract	142	10.0
Self-employed/Unemployed/Retired	364	25.6
Others	95	6.7
	**Mean**	**SD**
**Number of children**	1.2	1.0
**Number of children aged above 15**	0.4	0.7
**Age**	35.0	11.0

The history of exposure to COVID-19 is described in **Table 3**. We found that 2.4% (n = 8) and 7.0% (n = 24) of participants had COVID-19 confirmation tests and were isolated within the last two weeks. Overall, 10.8% (n = 43) of participants reported having a history of exposure to COVID-19, in which 4.7% (n = 16) had close contact with individuals suspected of COVID-19.

**Table 3:** History of exposure of COVID-19 of participants*.

Characteristics	n	%
**Having COVID-19 confirmation test in the last 14 days**		
Yes	8	2.4
No	333	97.7
**Being isolated in the last 14 days**		
Yes	24	7.0
No	317	93.0
**History of exposure to people with COVID-19**		
Close contact with people withCOVID-19	3	0.9
Indirect contact with people with COVID-19	15	4.4
Close contact with people suspected of having coved-19	16	4.7
Touching objects with the possibility of spreading SARS-CoV-2 (utensils)	9	2.6
Never having contact (directly or indirectly) with people with COVID-19	304	89.2

*Only 341 cases were reported.

The psychological impacts of the COVID-19 pandemic measured by IES-R are shown in **Table 4**. The mean score of the IES-R scale was 17.5 (SD = 11.5) (score ranged 0–88). Of all participants, 233 (16.4%) reported low level of PTSS; 76 (5.3%) rated as probably having moderate PTSS; 77 (5.4%) reported extreme conditions. The mean score of the intrusion subscale was the highest [8.9 (SD = 5.6) and score ranged from 0 to 32], followed by avoidance [4.4 (SD = 4.3) and score ranged from 0 to 32] and hyperarousal [4.3 (SD=3.8) and score ranged from 0 to 24).

**Table 4:** Psychological impacts (IES-R score) related to COVID-19 of participants.

Characteristics	n	%
**IES-R score interpretation**		
Normal	1,037	72.9
Low PTSS (PTSD clinical concern)	233	16.4
Moderate PTSS (Probable PTSD diagnosis)	76	5.3
Extreme	77	5.4
	**Mean**	**SD**
**Intrusion (Score range: 0**–**32)**	8.9	5.6
Any reminder brought back feelings about it	2.3	1.0
I had trouble staying asleep	0.9	1.0
Other things kept making me think about it	1.3	1.0
I thought about it when I did not mean to	0.8	0.9
Pictures about it popped into my mind	1.6	1.1
I found myself acting or feeling like I was back at stressful life events	0.7	0.9
I had waves of strong feelings about it	1.1	1.0
I had dreams about it	0.2	0.5
**Avoidance (Score range: 0**–**32)**	4.4	4.3
I avoided letting myself get upset when I thought about it	1.0	1.0
I felt as if it had not happened or wasn’t real	0.4	0.9
I stayed away from reminders of it	0.4	0.7
I tried not to think about it	0.6	0.9
I was aware that I still had a lot of feelings about it, but I did not deal with them	0.5	0.8
My feelings about it were kind of numb	0.4	0.7
I tried to remove it from my memory	0.5	0.8
I tried not to talk about it	0.5	0.8
**Hyperarousal (Score range: 0**–**24)**	4.3	3.8
I felt irritable and angry	0.6	0.9
I was jumpy and easily startled	0.6	0.9
I had trouble falling asleep	0.5	0.8
I had trouble concentrating	0.6	0.8
Reminders of it caused me to have physical reactions	0.2	0.6
I felt watchful and on-guard	1.7	1.2
**IE** **S-R score (Score range: 0**–**88)**	17.5	11.5

Cronbach’s alpha of domains Intrusion, Avoidance, Hyperarousal, and IES-R scale were 0.87; 0.80; 0.80; and 0.91, respectively.

**Table 5** depicts the factors associated with the psychological impacts caused by the COVID-19 pandemic among participants. Participants who were female compared to male (OR = 1.24; 95%CI = 1.01–1.61) or those who were above 44 years old compared to under 25 years old (OR = 3.70; 95%CI=1.89–7.25) had higher odds of having a greater score of IES-R. In addition, one higher number of children in the family was associated with 1.68 higher odds (95%CI = 1.31–2.16) to have a higher level of psychological impacts as measured by the IES-R scale. Having an educational level as postgraduate compared to high school or being self-employed/unemployed/retired compared to functionary was positively related to a higher score of intrusion and hyperarousal subscale. Participants who were white-collar workers compared to health care workers or having a history of touching objects with the possibility of spreading SARS-CoV-2 (utensils) compared to not having, also had a higher score on the subscale of avoidance.

**Table 5:** Factors associated with the psychological impacts of participants.

	IES-R score level		Score					
			Intrusion		Avoidance		Hyperarousal	
	OR	95% CI	Coef.	95% CI	Coef.	95% CI	Coef.	95% CI
**Gender** (Female vs. Male)	1.24**	1.01; 1.61					0.40	−0.10; 0.90
**Region** (Central vs Northern)	2.51**	1.18; 5.36					1.32*	−0.16; 2.79
**Age group** (vs Under 25)								
25–34	1.08	0.55; 2.12	−2.47***	−4.20; −0.74	0.08	−1.06; 1.23	−0.53	−1.40; 0.33
35–44	1.93*	0.98; 3.81	−2.18**	−4.24; −0.13	1.57**	0.22; 2.92	−0.22	−1.27; 0.83
Above 44	3.70***	1.89; 7.25	−1.12	−3.20; 0.95	2.37***	1.00; 3.74	0.88	−0.19; 1.96
**Marital status** (*vs* Single)								
Living with a spouse/partner			1.29*	−0.00; 2.58	−0.65	−1.63; 0.33	1.47***	0.63; 2.30
**Education level** (*vs* High school and below)								
Undergraduate			1.96	−0.53; 4.46			2.30***	0.78; 3.82
Postgraduate			5.87***	2.35; 9.39			2.73***	0.94; 4.52
**Occupation** (*vs* Health workers)								
Professional educators			−1.97*	−4.27; 0.33			−1.97*	−4.27; 0.33
White-collar workers					1.95***	0.49; 3.41		
Others					2.25***	0.61; 3.89		
**Occupation status** (*vs.* functionary)								
Limited-term full-time contract	2.16*	0.98; 4.74						
Self-employed/Unemployed/Retired			2.64**	0.03; 5.24			1.97***	0.47; 3.46
Others			−3.39**	−6.20; −0.57	−1.85	−4.23; 0.52	−2.00**	−3.77; −0.24
**History of exposure to people with COVID-19** (Yes *vs* No)								
Close contact with people with COVID-19	4.74	0.59; 7.91						
Touching objects with the possibility of spreading SARS-CoV-2 (utensils)					4.17**	0.26; 8.07	2.11	−0.79; 5.02
**Number of children**	1.68***	1.31; 2.16	1.14**	0.22; 2.06			0.82***	0.31; 1.33

***p < 0.01, **p < 0.05, *p < 0.1.

## Discussion

Findings from our study suggest critical evidence of initial negative psychological responses to the first time of large-scale social isolation among the general public. More than one-fourth of participants experienced mild to severe PTSS. The intrusion was rated with the highest score, which presented that people may have intrusive thoughts or nightmares about the problems. Being female, above 44 years old, or having a higher number of children in the family was associated with increased odds to a higher level of psychological impact. Additionally, being self-employed/unemployed/retired compared to functionary led to a higher odds of a clinically significant score of intrusion and hyperarousal subscale. Individuals who had a history of touching objects, with the possibility of spreading coronavirus (utensils), had greater odds of a higher level of avoidance.

This study shows relatively high rates of participants suffering from PTSS during the national lockdown related to COVID-19. This result is higher than the findings of studies that assessed post-traumatic stress (PTS) symptoms of hospital employees in China (29) and psychological impacts of the Taiwan population during the post-SARS epidemic in 2003 (29, 30). Another study carried out in the hardest-hit Hubei province, China, found substantially lower rates of PTSS using the PTSD Checklist for DSM-5 (PCL-5) (31). However, our findings showed a lower rate of PTSS compared to China’s general public (13), general workforce (32), psychiatric patients (33), and healthcare workers (34) during the COVID-19 pandemic based on the IES-R tool. Two studies on 52,730 Chinese respondents (35) and 18,147 Italian individuals (36) revealed a higher percentage of the psychological problems, notwithstanding differences in assessment tools. The number of COVID-19 cases and fatalities continues to increase in Europe compared to Vietnam, which may trigger more devastating consequences to health and the economy. The differences regarding measure instruments and sample size should also be considered. The impact of COVID-19 is intense and has the potential to be far greater than that of the Severe Acute Respiratory Syndrome (SARS) in 2003. The COVID-19 pandemic has dominated headlines around the world. In many counties, social distancing will not be lifted shortly, which may have more severe psychological impacts among the public.

Our study result is consistent with previous research in China, which showed that female participants had a higher risk of mental health outcomes (31, 35). The result suggests more prevalent acute psychological disorders after traumatic events characterized by more intrusive memories in females than males (37). The altered sensitivity to emotional stimuli among women can be attributable to differences in immune function and hormone levels, which may increase intrusive flashbacks and psychological disorders in females (38). Also, having a higher number of children in the family was positively related to more severe PTS, which can be explicated by economic harms and loss of parental productivity due to increased time of taking care of children at home during social isolation, especially in low-income families (39, 40). Moreover, during school closure, the higher number of children is associated with the increased time that women have to spend in unpaid care work. Studies show that women may face a double burden—having shifts at work and taking care of family members at home (41, 42). Therefore, the pandemic has shed light on the gender inequalities in the already existing global care crisis.

Individuals above 44 years old were more likely to experience a higher IES-R score than those under 25 years old. Middle-aged adults are also high-risk groups of COVID-19 due to the start of decreasing function, chronic underlying diseases that may weaken the immune systems (43). Also, self-employed/unemployed/retired participants had a higher score of intrusion and hyperarousal. The economic consequences of the COVID-19 pandemic characterized by reductions in working hours and wages push millions of people into unemployment, underemployment, and working poverty (8). Many companies have been forced to scale down their production or temporarily suspend operations amid the COVID-19 crisis (44). The reduced operations have led to job loss among self-employed workers in developing countries (44). Unforeseen financial pressures and decreased access to food are risk factors for psychological disorders and severe socio-economic distress (45, 46).

Having a history of touching objects with the possibility of spreading SARS-CoV-2 (utensils) related to a higher level of avoidance, which is consistent with a previous study (13). As using shared plates and bowls during their mealtimes is a ritual of Vietnamese people, those who share utensils are more likely to have a fear of contracting COVID-19. Experiences from the SARS epidemic in 2003 suggested that enhancing perceptions of people towards precautionary measures can lead to positive psychological responses by providing them with a sense of control in prevention (47).

Several implications can be drawn from the study. First, it is critical to set up comprehensive strategic planning to screen psychological problems and epidemiological monitoring, especially among the high-risk groups for early interventions. Second, programs are needed that focus on building women’s resilience to alleviate the care burden and better redistribute unpaid care work between women and men. Third, it highlights the need for more significant support for middle-aged adults and the elderly, affected laborers with psychological interventions to address distress and social concerns during these difficult circumstances. Fourth, regarding health care professionals, psychological support could include counseling services and assistance hotline teams to promote support systems among colleagues.

This study has several strengths, including large sample size and conducting in the first national lockdown, which becomes a piece of evidence. Additionally, we used international instruments that can increase the ability to compare our findings with previous studies. However, there were some limitations. A snowball sampling technique may limit the representativeness of the Vietnamese population, as in this study, the percentage of female participants and those who were from the northern region were high. Therefore, high sample size and recruiting participants from various settings/provinces were applied to increase the generalizability of results. Relying on the web-based platform, re-sharing the link could introduce selection bias with high uniformity. The cross-sectional study may limit the causal interpretation, and a longitudinal study on the psychological impact on Vietnamese is required (48).

In conclusion, there were relatively high rates of participants suffering from PTSS during the first national lockdown related to COVID-19. Being female, above 44 years old, having a higher number of children in the family, or being self-employed/unemployed/retired were associated with greater odds of having increased psychological distress. Comprehensive strategic planning to screen for psychological problems and appropriate interventions is critical, especially among the high-risk groups.

## Data Availability Statement

The raw data supporting the conclusions of this article will be made available by the authors, without undue reservation.

## Ethics Statement

The studies involving human participants were reviewed and approved by The Review Committee of the Institute for Preventive Medicine and Public Health, Hanoi Medical University. The patients/participants provided their written informed consent to participate in this study.

## Author Contributions

All authors contributed to the article and approved the submitted version. Data analysis: HQP and LV. Methodology: AD, HBTP, TN, QP, NT, QD and QTN. Supervision: HL, TD, AN, BT, CL, CH and RH, Writing-original draft: AD and XL. Writing -review and editing: AD, JT, QNN, MH.

## Funding

Research is supported by Vingroup Innovation Foundation (VINIF) in project code VINIF.2020.Covid-19.DA03.

## Conflict of Interest

The authors declare that the research was conducted in the absence of any commercial or financial relationships that could be construed as a potential conflict of interest.
